# Aversive emotion rapidly activates orexin neurons and increases heart rate in freely moving mice

**DOI:** 10.1186/s13041-021-00818-2

**Published:** 2021-06-30

**Authors:** Akira Yamashita, Shunpei Moriya, Ryusei Nishi, Jun Kaminosono, Akihiro Yamanaka, Tomoyuki Kuwaki

**Affiliations:** 1 Department of Physiology, Kagoshima University Graduate School of Medical and Dental Science, Kagoshima, 890-8544 Japan; 2 Department of Neuroscience II, Research Institute of Environmental Medicine, Nagoya University, Nagoya, 464-8601 Japan

**Keywords:** Aversive emotion, Stress, Orexin, Defense response, Fiber photometry

## Abstract

The perifornical area of the hypothalamus has been known as the center for the defense response, or fight-or-flight response, which is characterized by a concomitant rise in arterial blood pressure, heart rate, and respiratory frequency. It is well established that orexin neurons, which are located in this region, play a critical role in this response. In this study, we further examined this role by recording orexin neuronal activity and heart rate in freely moving mice using an original dual-channel fiber photometry system in vivo. Analysis of orexin neuron activity in relation to autonomic responses to aversive stimuli revealed a rapid increase in neuronal activity just prior to changes in heart rate. In addition, we examined whether orexin neurons would be activated by a conditioned neutral sound that was previously associated with aversive stimulus. We show that the memory of the aversive stimulus activated orexin neurons and increased heart rate. Our data suggest that orexin neurons are a key component linking aversive emotions to autonomic defense response. Our data also suggest that targeting orexin neurons may enable treatment of psychiatric disorders associated with chronic stress and traumatic memories.

## Introduction

Stress is often mistakenly thought of as being purely detrimental. While it is true that excessive stress generally has negative effects that can cause various mental disorders and emotional disturbances [1], responses to acute stress can be beneficial and even indispensable for life because they protect against potential sources of danger. Acute stress induces a rise in blood pressure, heart rate, respiration, and stress-induced analgesia. These autonomic changes are collectively called the defense response and prepare for and support fight-or-flight behavior [2]. We previously found that orexin-producing neurons in the hypothalamus play a crucial role in this defense response because it is severely attenuated in orexin knockout mice [3] and orexin neuron ablated mice [4].

Although orexin neurons are essential to autonomic defense responses induced by stress in general, little is known about their neuronal activity in response to specific stressors. In the past, several technical limitations have prevented us from thoroughly examining these unknowns. First, the use of freely moving animals without anesthesia is necessary because stress responses are unable to occur when anesthetized. Second, the hypothalamus contains many different cell types and targeting only the orexin-producing neurons of interest proved difficult. Some researchers have reported on the activity of orexin neurons in awake rats by using traditional electrophysiological methods such as extracellular recording and subsequently identifying the neurons that were recorded from with immunohistochemistry [5, 6]. However, this is a time-consuming approach and therefore is not realistic to use for studying the possible effects of several kinds of stressors while simultaneously recording autonomic stress responses. In addition, single-cell recording techniques create a sampling bias: there are about 2000–3000 orexin neurons in the mouse brain [7] but the activity of only one or two neurons can be recorded at a time. To overcome these limitations, we set out to develop and improve a fiber photometry system.

A fiber photometry system can track the real-time dynamics of genetically specified neuron populations located deep within the brain of freely moving mice by using a single channel fiber and genetically encoded Ca^2+^ indicators [8–10]. We recently utilized a single channel fiber photometry system and successfully recorded orexin [11, 12] and dopamine [13] neuronal activity using G-CaMP6. More recently, we upgraded the system to operate via dual channels. The dual-channel system can detect more sophisticated data via two-color measurement(s) by using one color for reference. Animal movement sometimes has an effect on the fluorescent signal intensity so this improvement was essential for the simultaneous recording of neuronal activity and animal behavior. We used the mCherry signal as a reference to analyze the G-CaMP signal more accurately. Although we briefly reported on this dual channel system in our previous paper [12], it will be described in more thorough detail here. The dual-channel system has previously been described by others [14, 15]. However, our fiber system has no joints between the region being recorded and the photodetector. Thus, the light transmission efficiency of the blue light excitation in our single fiber system is theoretically higher than the ferrule systems already published that include one joint. Recently, some groups have published multi-fiber photometry systems that use bundled fibers [16, 17]. Bundling very thin fibers provides better spatial resolution than our single fiber system and sometimes it seems that the activity of individual cells can be recorded. However, since the fiber is very thin, it is expected that the capability to detect smaller changes in fluorescence is lower.

Stress activates limbic structures and triggers emotional responses that are manifested in the body via an autonomic response. That is, stressors form emotions in the brain, which are then transmitted to the whole body by the autonomic nervous system. It has been shown that some orexin-producing neurons are involved in this pathway. However, the detailed time-course of the relationship between orexin neuronal activity and the autonomic response at the moment of stress remains unclear. Examples are fear-conditioning from social defeat [18], electric shock [19], and mate vocalization [20]. It is interesting to point out that previous research showed that orexin neurons contribute more preferentially to psychological stressors than to physical stressors [21].

Furthermore, since orexin neuron activity is linked to arousal [5, 6], there is a problem in distinguishing between arousal-related and stress-related autonomic changes. This is because the experimental stress is accompanied by an arousal component via the sensory system. Pavlovian aversive conditioning, on the other hand, can turn an innocuous neutral sound with no arousal component into a stimulus that evokes aversive emotions. Comparing responses to such sound before and after aversive conditioning will give us an insight into emotion-related changes because of the absence of an arousal component.

To clarify the activity of orexin neurons at the moment of stress and to reveal a possible relationship between orexin neuronal activity and the heart rate response, we measured orexin neuronal activity induced by aversive stimuli using a dual-channel fiber photometry system and heart rate using a telemetry system in freely moving mice. In addition, we attempted to clarify the possible effect of orexin neuronal activity in the case of acquired aversive emotions after conditioning.

## Results

### Setup of dual-channel in vivo fiber photometry system

**Fig. 1:**
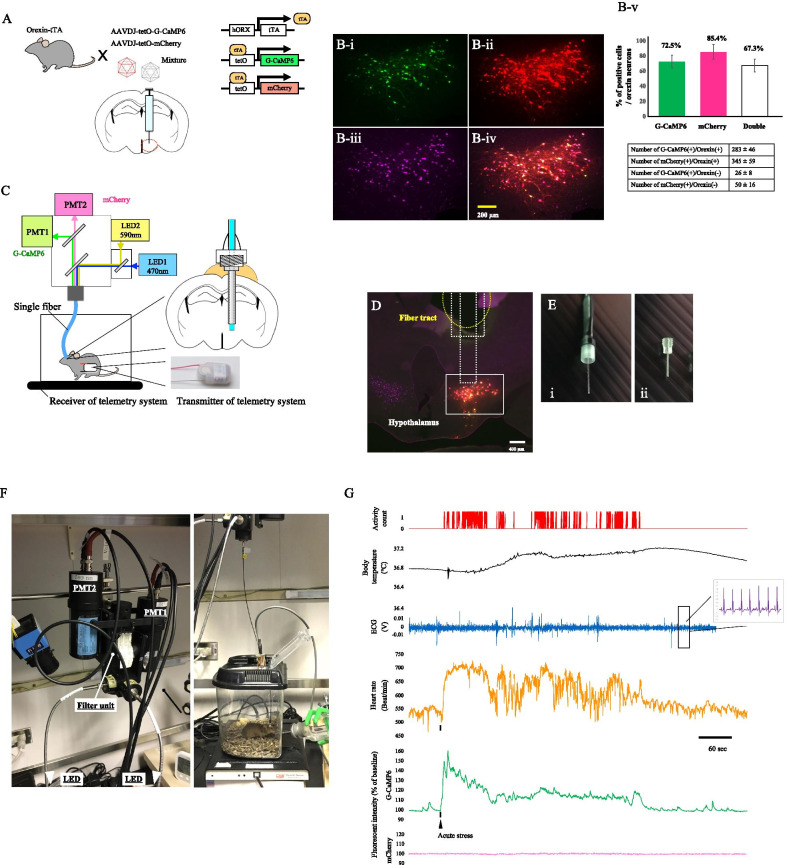
Simultaneous real-time measurement of orexin neuronal activity, heart rate, and body temperature in freely moving mice using a fiber photometry and telemetry system. **A** Schematic drawing showing specific expression of G-CaMP6 and mCherry in orexin neurons by injecting AAV vectors into the hypothalamus of Orexin-tTA transgenic mice. **B** Immunohistochemical confirmation showing G-CaMP6 (**B-i**) and mCherry (**B-ii**) exclusively expressed in almost all orexin neurons (**B-iii**) that were visualized by anti-Orexin A-antibody in Orexin-tTA mice. G-CaMP6 and mCherry were identified by their own fluorescence. A composite image is shown in (**B-iv**). Images are a close-up of the white rectangle in **D**. **B-v** Quantification of G-CaMP-positive cells, mCherry-positive cells, and G-CaMP6 and mCherry double-positive cells in hypothalamic orexin neurons. **C** Schematic diagram of the dual-channel fiber photometry system. Blue excitation light from the 470 nm LED illuminates the G-CaMP6-expressing neurons in the hypothalamus via optical fiber. Green fluorescence from G-CaMP6 is then gathered by the same optical fiber and detected by the photomultiplier tube (PMT1). Body temperature and heart rate were measured by a telemetry system. The telemetry transmitter was intraperitoneally implanted into the mouse. **D** Confirmation of the fiber tract after fluorescent recordings. Coronal section of the brain from the orexin-tTA mouse injected with AAV-tetO(3G) G-CaMP6 and AAV-tetO(3G) mCherry mixture 2 weeks before recording. The dashed line indicates the location of the inserted guide cannula and optic fiber. The white square indicates the hypothalamus region. **E** Self-assembled silica connecting cable called the “patch cord” (i) and in vivo optical fiber cannula (ii). **F** Overall fiber photometry system. **G** Representative traces, in response to ultrasonic sound stress, of locomotor activity (activity count), body temperature, electrocardiography (ECG, expanded traces in the rectangle), heart rate (calculated as beats per minute), and the fluorescence intensity of G-CaMP6 and mCherry in the orexin neurons

Fiber photometry systems can track the real-time dynamics of genetically specified neuronal populations in the deep brain of freely moving mice by using a single fiber and genetically encoded Ca^2+^ indicators [9]. We improved this system by adding a second channel to be able to simultaneously detect both G-CaMP6 and mCherry. To specifically express G-CaMP6 and mCherry in orexin neurons, we prepared transgenic mice and AAV vectors as shown in Fig. 1A. The AAV mixture consisting of AAVDJ-tetO-G-CaMP6 and AAVDJ-tetO-mCherry was stereotaxically injected into the hypothalamus of ORX-tTA mice that express tetracycline transactivator (tTA) in orexin neurons (Fig. 1A). These AAVs can drive expression of G-CaMP6 and mCherry proteins in the presence of tTA. Three weeks after this mixture was injected, G-CaMP6 and mCherry expressed almost exclusively in hypothalamic orexin neurons (Fig. 1B). In 393 ± 60 orexin positive neurons (n = 6 animals), G-CaMP6(+) cells were 283 ± 46, mCherry(+) cells were 345 ± 59, and both G-CaMP6− and mCherry-expressing orexin neurons were 260 ± 43 (Fig. 1Bv). There was a small amount of ectopic expression of G-CaMP6 (G-CaMP6(+) and orexin (−) out of total G-CaMP6(+) was 8.3%). Blue and yellow excitation lights for G-CaMP6 and mCherry were supplied by LEDs and delivered through a single optical silica fiber (Fig. 1C). Fluorescence emission from G-CaMP6 and mCherry expression in the hypothalamic orexin neurons pass back through the single optical silica fiber. The fluorescent signals pass through two dichroic mirrors and separate into green fluorescence derived from G-CaMP6 and red fluorescence derived from mCherry. The signals then arrive at their respective photomultipliers (Fig. 1C). To place the single optical silica fiber in the desired region of the hypothalamus, we implanted a guide cannula with dental cement (Fig. 1C). The guide cannula allowed the optical fiber to be placed just dorsal to the hypothalamic area of interest (Fig. 1D). The single optical silica fiber and guide cannula were made in our laboratory with the support of LUCIR, Inc. (Tsukuba, Ibaraki, Japan) (Fig. 1E). These tools allowed us to obtain a stable, noiseless signal with high temporal resolution. To measure electrocardiogram (ECG) and body temperature, we implanted a telemetry system transmitter (Data Sciences International, St. Paul, MN, USA) into the abdominal cavity at the same time as the guide cannula implantation (Fig. 1C). The complete system is shown in Fig. 1F. This system allowed for several types of physiological data to be measured in freely moving mice receiving food and water ad libitum. The data was measured simultaneously under a high temporal resolution (Fig. 1G). G-CaMP6 signals responded independently from shifts in mCherry signals, so we could determine whether the G-CaMP6 signal was real by comparing it to the levels of mCherry fluorescence. The results shown in Fig. 1 demonstrate we were able to utilize a dual-channel fiber photometry system.

### Association between orexin neuronal activity and stress-induced autonomic responses

**Fig. 2:**
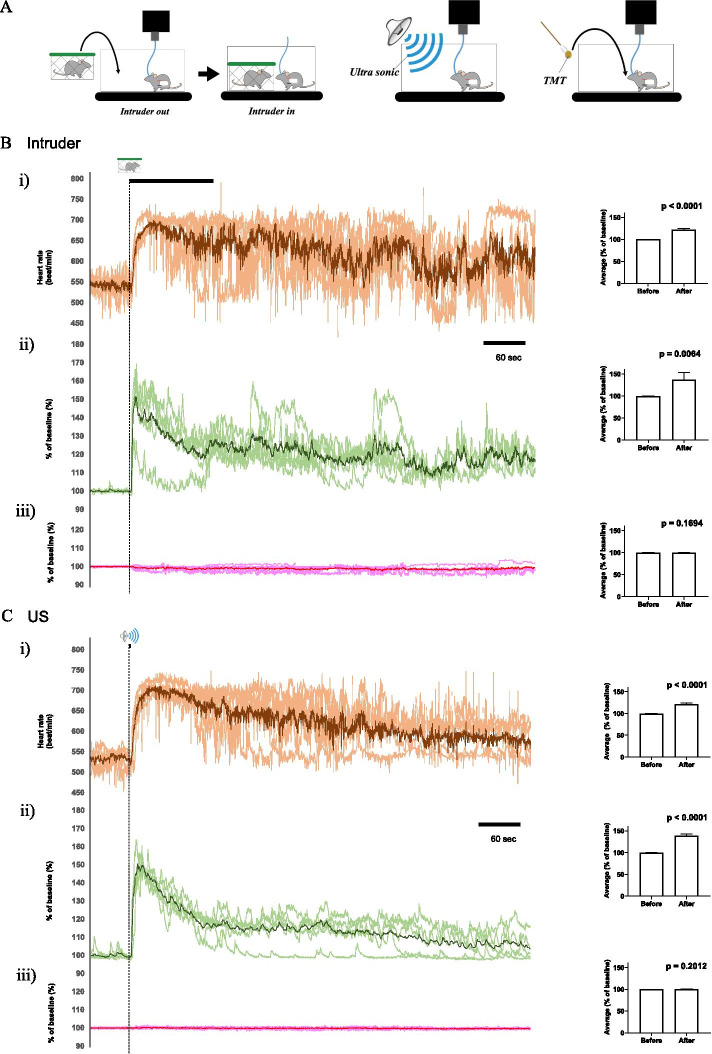

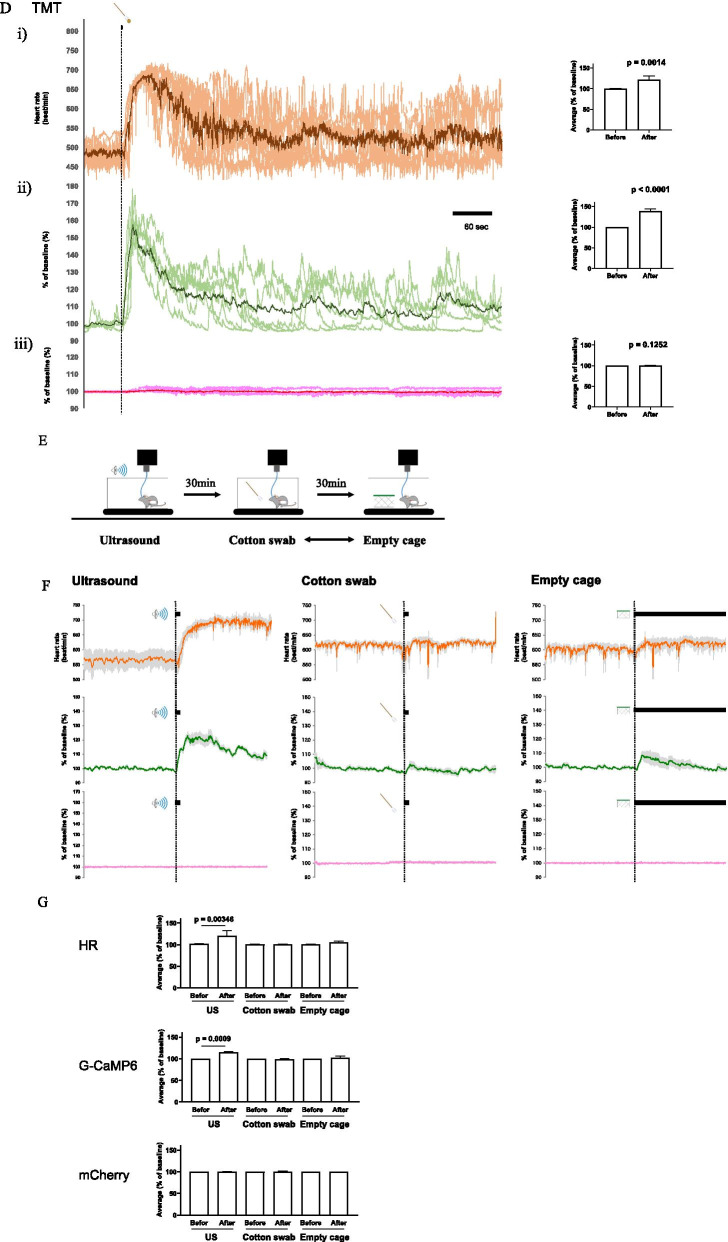
Aversive stimuli activated orexin neurons together with an increase in heart rate. **A** Schematic drawing showing the experimental design; intruder stress (left), ultrasound stress (middle), and predator odor (TMT) stress (right). **B** Results of intruder stress experiment. The horizontal bar shows the time when the intruder was in the experimental chamber for 2 min. (i) (Left) Averaged time course of the heart rate during the intruder test. The mean value is shown in the dark orange line and values of individual animals are shown in light orange. (Right) The bar graph shows averaged values of the heart rate for 1 min during the baseline period and after the introduction of an intruder. (ii) (Left) Averaged time course of the G-CaMP6 fluorescence during intruder test. The mean value is shown in the dark green line and values of individual animals are shown in light green. The horizontal bar shows the time when the intruder was in the experimental chamber. (Right) Bar graph shows averaged values of the intensity of G-CaMP6 fluorescence for 1 min during the baseline period and after the introduction of the intruder. (iii) Results of mCherry fluorescence are shown in a similar manner to **B-ii**. **C** Results of the ultrasound stress experiment. Data are shown in a similar manner to **B**. The horizontal bar shows the time when ultrasound was applied for 2 s. **D** Results of TMT smell experiment. Data are shown in a similar manner to **B**. The horizontal bar shows the time when TMT was applied for 2 s. Data were sampled in six animals in the intruder test and ultrasound stimulation and seven animals in the TMT test. Group data are presented as mean ± SEM. P-value was calculated using paired Student’s t-test. **E** Schematic drawing showing the control experimental design; mice (n = 4) were given ultrasound stress for positive control. Then odorless cotton swab and the empty cage were presented in intervals of 30 min. For randomization, 2 mice received cotton swab for the first time and the other mice received empty cage for the first time. **F** Traces of the heart rate (upper), G-CaMP6 fluorescence (middle), and fluorescence of mCherry (lower) in ultrasound stress group (left), cotton swab group (middle), and empty cage group (right). The colored line indicates averaged value and the gray line shows SEM. **G** (Upper) Bar graph showing averaged values of the heart rate for 1 min during the baseline period and after the introduction of an intruder. (Middle) Bar graph showing averaged values of the intensity of G-CaMP6 fluorescence for 1 min during the baseline period and after the introduction of intruder. (Lower) Results of mCherry fluorescence are shown in a similar manner to G-CaMP6. Group data are presented as mean ± SEM. P-value was calculated using paired Student’s t-test

To plot real-time orexin neuronal activity dynamics and autonomic response dynamics before and after various types of aversive stimuli, we simultaneously measured G-CaMP6 fluorescence and ECG. Heart rate was calculated from the ECG recordings. As an autonomic response to emotional stress, we analyzed heart rate response because a change in body temperature was slow when compared to change in heart rate (see Fig. 1G). Three different stress paradigms with different sensory modalities and different afferent pathways were used. The paradigms were intruder stress, aversive sound stress, and aversive smell stress (Fig. 2A).

All of the stimuli tested, namely intruder (Fig. 2B), ultrasound (Fig. 2C), and predator odor (Fig. 2D), induced abrupt increases in heart rate (Fig. 2B–D, upper) and G-CaMP6 fluorescence (Fig. 2B–D, middle), while mCherry fluorescence (Fig. 2B–D, lower) did not change. As a control for these stimuli, the physiological response was measured using a cotton swab without odor and an empty box without intruders (Fig. 2E). These stimuli did not increase G-CaMP6 fluorescence or heart rate as much as ultrasound stress (Fig. 2F, G).

**Fig. 3:**
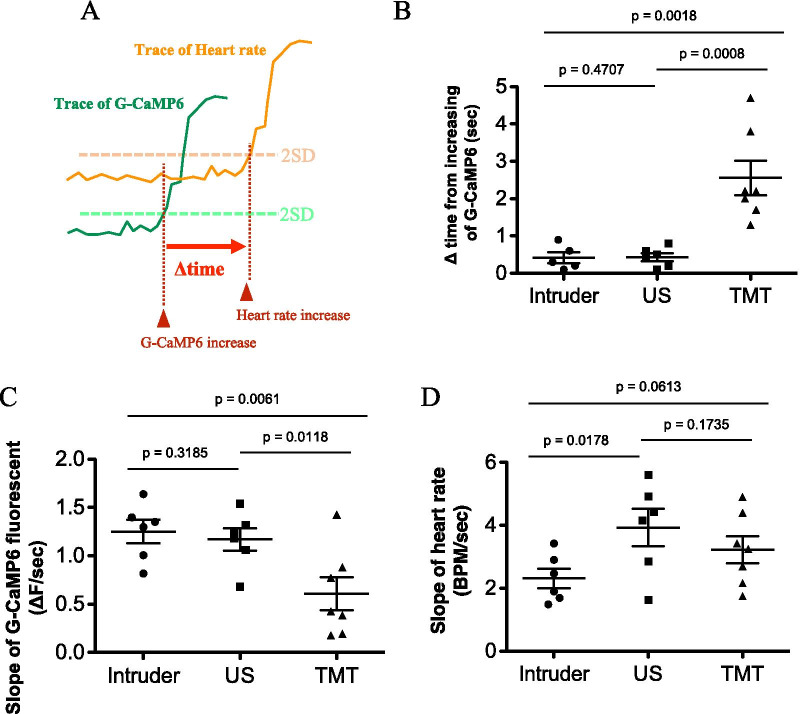
Comparison of onset latencies and increasing rate between the change in G-CaMP6 fluorescence and those in the heart rate. **A** Schematic drawing of the definition of ∆time. ∆time was defined as the difference between the onset of fluorescence change and the onset of the heart rate change. Onset was defined as the time when the signals first exceeded 2SD from the baseline fluctuation. **B** Plots of Δtime in the three stress experiments (intruder, ultrasonic sound; US, and TMT). Horizontal bars indicate the average value and SEM. One-way ANOVA indicated there was significant statistical difference among stresses (F(2,15) = 14.88, P < 0.0003). Tukey’s multiple comparisons was used as a post hoc test (P values in the figure). **C** Plots of the slope in G-CaMP6 fluorescence. The slope of the signal increase was defined as (maximum value − baseline value/time at maximum − time at the onset). Horizontal bars indicate the average value and SEM. One-way ANOVA indicated there was statistical difference between stresses (F(2,16) = 6.317, P = 0.0095). Tukey’s multiple comparisons was used as a post hoc test (P values in the figure). **D** Plots of the slope in heart rate trace. Horizontal bars indicate the average value and SEM. One-way ANOVA indicated there was no statistical difference among stresses (F(2,16) = 2.994, P = 0.0786). Data were sampled in 6 animals

Although increases in G-CaMP6 fluorescence and heart rate appeared to occur simultaneously (Fig. 2B–D), closer examination revealed that there were differences in the responses. To examine possible differences in the time course, we defined onset as the time when the signal more than doubled the standard deviation of the baseline fluctuation (Fig. 3A). We then calculated ∆time to be the difference between the onset of the increase in heart rate and the onset of the increase in G-CaMP6 signal. As a result, ∆time was a positive value in all the stimuli in every mouse (Fig. 3B) meaning that the change in orexin neuronal activity preceded the increases in heart rate in all events. It is of interest to point out that ∆time in the TMT test was significantly longer than in the intruder test and the ultrasound test while there was no difference between the intruder test and the ultrasound test, indicating non-uniform changes in heart rate depending on stress stimuli.

We also calculated the slope of the signal increase (maximum value − baseline value/time at maximum − time at the onset). This analysis revealed that the slope of G-CaMP6 fluorescence change in the TMT group was significantly smaller than that in the intruder and ultrasound groups (Fig. 3C). Meanwhile, the slope of the heart rate change did not differ among the three stimuli (Fig. 3D). For TMT stimulation, activation of orexin neurons was relatively gradual when compared to the other stimulation types, and the start of the heart rate response was delayed, but the increase in heart rate ended up being similar.

### Conditioned sound stimulation activated orexin neurons

Although the previous results have shown that activation of orexin neurons precedes heart rate increase thus indicating a close association between orexin neuronal activity and autonomic outcome, these experiments were limited to innate aversive responses.

To determine if this was also the case with acquired negative emotions, we evaluated the change in orexinergic neuronal activity resulting from exposure to a neutral tone that was previously conditioned as a cue for an aversive experience. This classical conditioning paradigm allows us to compare the response to an emotionally neutral sound with the response when that sound becomes emotionally meaningful. In our preliminary experiment, we identified a neutral sound condition that did not result in an autonomic response and used it as the conditioned stimulus. Thus, this experiment can determine whether orexin-producing neurons are also involved in the response to centrally generated aversive emotional responses rather than simply natural or innate aversive stimuli.

**Fig. 4:**
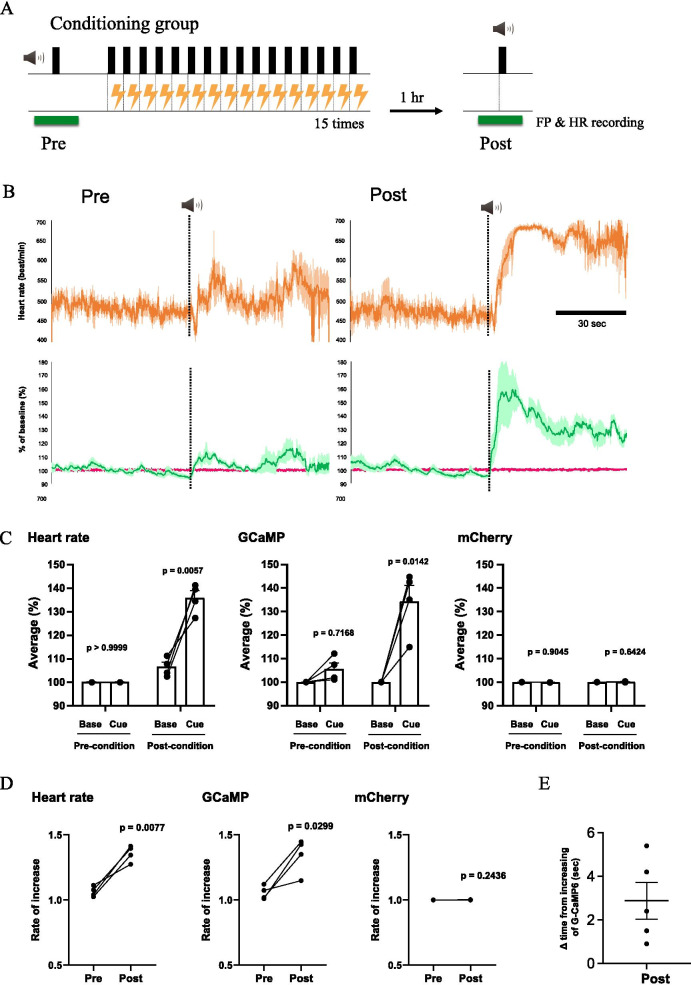

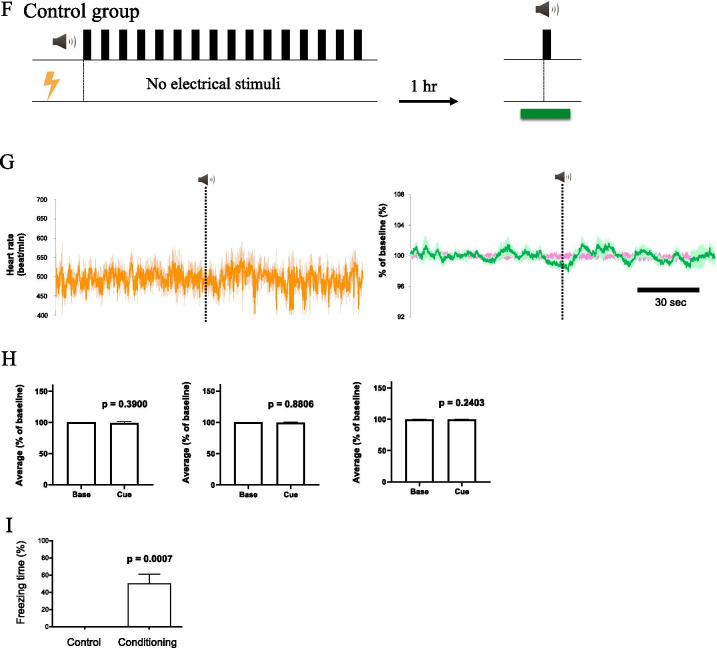
Conditioning-induced activation of orexin neuron and increase in heart rate without aversive sensory input. **A** Experimental design for neuronal and heart rate recordings of conditioning group (n = 4). Heart rate (HR) and fluorescence via fiber photometry (FP) were recorded for 2 min around sound application before conditioning (pre) and 1 h after conditioning (post). HR and fluorescence were averaged for 1 min during the baseline period and after the cue sound (green horizontal line). **B** Traces of the heart rate (upper), G-CaMP6 and mCherry fluorescence (lower) in pre-conditioning (left) and post-conditioning (right) in the conditioning group. The dark-colored line indicates the averaged value and the light-colored line shows SEM. **C** Grouped average and SEM of the averaged values for 1 min during the baseline period and after the cue sound. Two-way ANOVA revealed that there was significant difference between baseline and cue sound (Heart rate: F(1, 3) = 8.719, P = 0.0599; G-CaMP6: F(1,3) = 15.22, P = 0.0299; mCherry: F(1,3) = 2.096, P = 0.2435) and between pre-condition and post-condition (Heart rate: F(1, 3) = 622.9, P < 0.0001; G-CaMP6: F(1,3) = 30.91, P = 0.0115; mCherry: F(1,3) = 0.04808, P = 0.8405). P values in the figure were calculated by Bonferroni’s multiple comparison test. **D** Changes in the rate of increase due to the cue sound of individual mice. The rate of increase is represented by the average of after cue sound/the average of before cue sound. P values were calculated using a paired t-test. **E** The difference between the onset of fluorescence change and the onset of heart rate change. See Fig. 3A for the definition of ∆time. **F** Experimental design for neuronal and heart rate recordings in the control group (n = 11). **G** Traces of the heart rate (left), G-CaMP6 and mCherry fluorescence (right) in the control group. **H** Grouped average and SEM of the averaged values for 1 min during the baseline period and after the cue sound. P-values were calculated using paired t-test. **I** Results of the freezing score (% time) during cue test tone. In the post-session, freezing behavior was counted in the control group (n = 11) and in the conditioned group (n = 4). P-values were calculated using Mann–Whitney’s nonparametric test

First, while recording the orexin neuronal activity and electrocardiogram, we gave a neutral tone without electrical stimulation to the mouse (Fig. 4A). No significant changes in orexin neuronal activity and heart rate were observed after the sound exposure (Fig. 4B left, C). After conditioning was established as in the protocol shown in Fig. 4A, the mouse was exposed to the same sound that was applied in the preconditioning period (Fig. 4A). As a result, orexin neuronal activity and heart rate increased immediately and markedly (Fig. 4B right, C). Furthermore, we calculated sound-induced changes in heart rate and orexin neuronal activity and compared them before and after the conditioning (Fig. 4D). The rates of increase in heart rate and orexin neuronal activity were significantly higher after fear conditioning. ∆time was calculated and was a positive value in all the cue stimuli in every mouse as with Fig. 3B (Fig. 4E). This shows that the change in orexin neuronal activity preceded the increases in heart rate in all events with fear-conditioned mice. Conversely, orexin neuronal activity and heart rate did not change (Fig. 4G, H) when exposed to the neutral tone in the control conditioning protocol with no electrical stimuli shown in Fig. 4F. We examined whether conditioning was established by measuring freezing time during the observation period. As expected, conditioned group animals spent ~ 50% of the time in freezing while control animals did not (Fig. 4I).

## Discussion

In this study, we examined whether aversive stimuli and aversive emotions affect the activity of hypothalamic orexin neurons. We first constructed the recording system to assess neuronal activity from the specific cell type of interest deep in the brain of freely moving mice. Using in vivo recording of hypothalamic orexin neuronal activity and ECG, we showed that every examined stress type activated orexin neurons just before the change in heart rate took place indicating a potential causative relationship between orexinergic neuronal activity and the resulting autonomic outflow. In addition, we showed that the acquired aversive emotion in response to the neutral tone also activated orexin neurons. The autonomic response to aversive emotions, whether innate or acquired, was preceded by an increase in orexin neuronal activity.

Activation of orexin neurons in response to the acquired aversion of an otherwise emotionally neutral stimulus (conditioned situation) indicates a possible contribution of the orexin system in the etiology of psychiatric disorders resulting from chronic stress because symptoms may occur after the original sensory inputs no longer occur.

### The dual-channel fiber photometry system produces a good signal even in freely moving mice

Using our system, we were able to record and analyze neuronal activity and autonomic responses in freely moving animals with high temporal resolution (Fig. 1G). Two-color simultaneous recording allowed us to distinguish between signal and noise with confidence. Neuronal activity recording with head-fixed mice [11, 13] is an easy way to distinguish signal from noise because the animal’s head cannot move and affect the signal, but more natural behaviors cannot be measured. As a solution, measurements are taken via optical fiber from freely moving mice. However, in this case, the fiber may twist or move and the transmission efficiency at the connection between the fiber and the fluorescence detection device changes, and large amounts of noise may occur. The device shown in Fig. 1 was able to eliminate these kinds of noise issues. The fluorescence ratio of green and red may also be used when analyzing data, similar to ratiometry analysis employed when using a FRET system. However, we did not calculate the ratio and instead used the red signal as an indicator of stable recording. Although we were able to exclude unstable data that occurred during large or sudden animal movements as judged by the mCherry signal, it is necessary to determine a method to completely eliminate instability in future experiments. Criteria to exclude recordings was set to be 5% in F/F0 in mCherry fluorescence. No such change was observed in our experimental setting except for the electric foot shock period in the conditioning procedure. Fiber photometry systems also often struggle with the weakness of their signals. In previous papers, authors have tried to efficiently extract fluorescent signals with various methods such as time-correlated counting system, lock-in amplifier, averaging, and ratiometric measurement [8, 9, 22, 23]. We were able to solve this issue by implanting only a single fiber from the fluorescence detection device into the brain (Fig. 1E). Systems that use standard ferrules will commonly lose some fluorescence signals in their connections. The absence of such a joint is an advantageous feature of our system manufactured by LUCIR, Inc.

### Orexin neuronal activity and heart rate rapidly increase due to aversive stimulation irrespective of sensory modality

We showed here that orexin neuronal activity and heart rate are increased instantaneously by aversive stimuli in freely moving mice. Giardino et al. [24] already showed predator odor-induced activation of the lateral hypothalamic orexin neurons using fiber photometry. Unfortunately, however, they did not assess any possible autonomic consequences of orexinergic activation. We simultaneously recorded heart rate and tried three kinds of stressors to induce aversive emotion and mice showed similar responses to each stimulus (Fig. 2). It has already been shown that increased heart rate due to intruder stress is severely attenuated in orexin deficient mice [3, 4]. Therefore, orexin may be involved in the regulation of stress-induced autonomic responses. Furthermore, orexin neurons have dense innervation from the bed nucleus of the stria terminals (BNST) and the central nucleus of the amygdala (CeA) [25]. A recent study revealed that optogenetic/chemogenetic activation of GABAergic neurons in the BNST induces a rapid transition from NREM sleep to wakefulness through inactivation of melanin-concentrating-hormone neurons (which are located in the same hypothalamic region as orexin neurons [26]) and activation of orexin neurons [27]. A previous study has also shown that stress-induced autonomic responses are attenuated by pharmacological suppression of the activity of the BNST and CeA in wild-type mice [28]. The central medial nucleus of the amygdala (CeM) and BNST are known as areas that regulate emotion-related autonomic responses [28]. Therefore, it is suggested that when emotional stress is given to mice, the activity of the CeM and BNST is enhanced, the orexin neurons are subsequently activated, and the autonomic response will occur [2, 28]. In addition, inhibitory corticotropin-releasing factor-expressing BNST neurons seem to be directly connected to orexin neurons which suggests a complex network behind stress-induced orexinergic activation [24].

Three types of aversive stimuli were chosen for this study because we hypothesized that if the sensory component of stress directly activates orexin neurons, there might be a different response depending on the sensory modality. However, as shown in Fig. 2, all three stress types similarly activated orexin neurons even though the heart rate response from the TMT test was slightly different from that in the intruder test and ultrasonic test. This difference may be a result of the olfactory input circuit in the brain being unique. For example, it sends information directly to the limbic system without going through the thalamus-cortical circuit, unlike the other sensory circuits.

### A possible causal relationship between orexin neuronal activity and heart rate increase

When focusing on the differences between the starting point of the orexin neuronal activity increase and the starting point of the heart rate increase, we found that the onset of the increase in orexin neuronal activity always preceded changes in heart rate (Fig. 3B). Due to this finding, it seemed reasonable to conclude that there might be a causal relationship between them, namely that orexin neuronal activity may directly cause an increase in heart rate. Although our data did not fully prove causality, it is in line with pharmacological studies with orexin receptor antagonists and genetic studies with knockout mice (see Carrive and Kuwaki [28] for review). Current results provide a higher time resolution and strongly support these previous findings.

### Differences in orexin neuronal activity depending on the type of stress stimulation

Interestingly, as shown in Fig. 3B, the Δtime is only high in the group exposed to TMT. We further examined the difference in the rate of signal increase between the three types of stressors by examining the G-CaMP6 slope. This examination shows that the G-CaMP6 fluorescence intensity increase in the TMT group is relatively moderate, that is, the neuronal activity seems to rise slowly (Fig. 3C). However, the slope of the heart rate increase in the TMT group is the same as that in the other groups (Fig. 3D). Due to these findings, we hypothesized that orexin neuronal activity needs to reach a threshold to increase heart rate. Taken together, the Δtime in G-CaMP6 slope was prolonged because the TMT group took longer than the other groups to reach the orexin neuronal activity threshold required to initiate heart rate increase. We do not currently have an explanation for the reasons behind why the orexin neuronal activity rises slowly only in the TMT group. It may be that olfactory information differs from visual and auditory information in that it takes longer for the chemical substance to diffuse and bind to the receptor, and that the experimental result reflects the time difference from when the TMT stress is given until the mouse processes and interprets the odorous substances. We cannot deny the possibility that the heart rate response was mediated by some other neuronal system in parallel to orexin that contributes to the activation of heart rate. Such parallel systems may be differently affected by different aversive stimuli.

### The negative emotional component of stress activates orexin neurons and increases heart rate

Sensory input usually increases vigilance levels in animals regardless of the valence of emotion. The orexin neuronal activity we observed (Fig. 2) may be due to an increase in awareness that accompanies an increase in attention. We were unable to distinguish whether the result of the stress-induced increases in orexin neuronal activity and the subsequent autonomic responses shown in Fig. 2 were caused by an increased vigilance caused by sensory input alone or by an “emotional change” accompanying the sensory input. Therefore, we performed an experiment to distinguish between them by utilizing a fear-conditioning paradigm. Observation of freezing behavior clearly showed that the neutral sound did not cause any aversive emotional changes unless the sound was associated with electric shock (Fig. 4I). We observed an immediate increase in orexin neuronal activity and heart rate response only when the neutral tone was previously associated with aversive emotion (Fig. 4C). This observation is in line with reports showing a possible relationship between orexin and fear behaviors [21, 29, 30]. Considering the results of Fig. 4 and the description in the previous discussion, it is possible that the changes in orexin neuronal activity observed in Fig. 2 might depend on changes in the emotion of the animals, and not the individual sensory stimulation itself.

### Role of the orexin system in aversive emotion processing

In our previous study [11], we showed that orexin neurons were activated by painful stimuli (nociceptive stimuli). At that time, it was not possible to distinguish whether the response was caused by the ascending pathway from the nociceptive receptors or if it was caused by the descending pathway after the brain recognized the signal as painful. Based on the present study, we speculate that the affective component of the pain associated with the painful stimulus could also activate a descending pathway to the orexin neurons, independently of a direct ascending pathway from nociceptors.

Sears et al. [25] showed previously that emotional memory formation is an essential signal to the series circuit of the hypothalamus, locus coeruleus (LC), and the amygdala. Orexin activates OXR1 in the LC, which activates the amygdala that is involved in threat learning formation. Orexin neurons can be activated by cue stimulation once a threat memory has been associated with this cue. Therefore, it is inferred that the hypothalamus–LC–amygdala loop circuit is activated by cue stimulation during and after fear conditioning. On the other hand, the orexin system was rapidly activated even when an innate unpleasant stimulus was presented instead of an acquired fear cue. Aversive emotions activate the hypothalamus–LC–amygdala circuit to enhance threat learning and, at the same time, activate the fight or flight system to trigger an autonomous response (stress defense response). An immediate increase in orexin neuron activity due to aversive feelings may be a critical factor that simultaneously promotes several physiological responses relating to stress (e.g., anxiety, emotional memory formation enhancement, and sympathetic nerve activation).

## Conclusions

This paper shows that the use of LUCIR’s fiber photometry system with our modification will give us a reliable signal even when the animal is actively behaving. This system can easily be used in combination with ECG, EEG, EMG recording, and video tracking. It can provide simultaneous monitoring and analysis at a high temporal resolution of all the physiological pieces of information analyzed from neuronal activity deep in the brain. It was found that orexin neuronal activity is indeed increased due to aversive stimuli induced by the sensory modalities tested so far. The important finding is that the increase in orexin neuronal activity precedes the autonomic response. Aversive emotion serves a very important function as a warning system for keeping animals informed of a variety of dangers. Unfortunately, in many cases of modern society, repeated exposure to acute stress is unavoidable. For example, office workers may repeatedly suffer from aversive emotions, which may manifest in illnesses such as depression. While it may be difficult to completely escape from the stressor in these types of situations, controlling the orexin neuronal activity that mediates stress and autonomic responses may reduce the severity of the stress-induced disorders. These data suggest that targeting orexin neurons may enable prevention of psychiatric disorders that result from repeated acute stress.

## Methods

### Animals

We used transgenic mice carrying a tetracycline-controlled transactivator transgene (tTA) under the control of the orexin promoter [11] (orexin-tTA mice, n = 45). All experimental procedures were performed under the guiding principles for the care and use of animals in the field of physiological sciences published by The Physiological Society of Japan and were approved by the Institutional Animal Use Committee at Kagoshima University (MD15075, MD17090, MD18081). Mice were maintained under a strict 12-h light/dark cycle (light period: 7:00–19:00; dark period: 19:00–7:00) in a temperature-controlled room (22 °C). Food and water were available ad libitum and all efforts were made to minimize animal suffering and discomfort and to reduce the number of animals used.

In vivo recordings of neuronal activity using a fiber photometry system and cardiovascular parameters using a radio-telemetry system

### Stereotaxic AAV injection

Surgeries for AAV injections were conducted under isoflurane anesthesia (2%, inhalation) using a stereotaxic instrument (ST-7, NARISHIGE, Tokyo, Japan). A viral mixture consisting of recombinant AAV-tetO(3G)-G-CaMP6 (serotype: DJ; 600 nl/injection, 3 × 10^12^ copies/ml) and AAV-tetO(3G)-mCherry (serotype: DJ; 600 nl/injection, 6 × 10^12^ copies/ml) was stereotaxically injected into the right side the hypothalamic perifornical area (PeF) in orexin-tTA mice (Fig. 1A). All AAV used in this article were produced by Yamanaka’s Laboratory at Nagoya University, Japan (Methods of production and purification were described elsewhere [11]). The AAV mixture was injected via an air-pressure injection system (I-200J, NARISHIGE, Tokyo, Japan) connected by polyethylene tube to a glass micropipette that was made from a pulled glass tube (φ1.5 mm, World Precision Instruments, FL, USA) by a puller (Sutter Instrument Novato, CA, USA) and had a tip diameter of 18–22 μm. Injection site was as follows: from bregma anterior 1.5 mm, lateral 0.8 mm, ventral 5.0 mm from the dura.

### Implantation of optical fiber for fiber photometry system

Over 2 weeks after viral injection, mice were surgically implanted with a guide cannula (diameter; 600 µm, length of guide; 8 mm, made by LUCIR, Tsukuba, Japan) for placing the optical fiber just above the hypothalamus to record orexin neuronal activity (Fig. 1D). At the start of the surgical procedures, mice were anesthetized with isoflurane (2–3%, inhalation), and placed on a small animal stereotaxic instrument as described previously [11]. A holding fiber with the guide cannula jointed was attached to the stereotaxic instrument. The site of implantation for the guide cannula was as follows: from bregma anterior 1.5 mm, lateral 0.8 mm, ventral 5.0 mm from the dura. During implantation, the fluorescence signal was continuously monitored so that the optimal position of the fiber tip was easily recognized by an abrupt increase of the output signal. The guide cannula was fixed with dental cement (Fuji LUTE BC, GC, Tokyo, Japan), gel-type quick drying glue (LOCTITE 454, Henkel Japan, Yokohama, Japan), and a small anchor screw. After fixation of the guide cannula, the holding fiber was removed from the guide cannula and a dummy fiber was inserted to prevent dust from entering the implanted guide cannula during the recovery period.

### Implantation of the transducer for the radio-telemetry system

Immediately after performing the guide cannula implantation, an additional implantation surgery was performed on the mice. To measure heart rate and body temperature, we used a radio-telemetry system (Data Sciences International, St. Paul, MN, USA). This system consists of a radio-frequency transducer (TA11PA-C20) and a receiver (RLA1020). The body of the transmitter was implanted into the abdominal cavity. The paired insulated leads from the abdominal cavity were placed subcutaneously in the chest. Using a non-absorbable suture, the positive electrode (+) of the lead, which was placed in the left abdomen below the heart, was anchored to the inside of the skin. The negative electrode (−) of the lead, which was placed in the right upper chest, was anchored to the skin. Subsequently, the incision in the skin and peritoneum were sutured. A temperature sensor in the body of the transmitter allowed the abdominal temperature to also be measured. During all surgeries, care was taken to maintain body temperature. After surgeries, mice were treated with penicillin and an analgesic, buprenorphine. For recovery, mice were individually housed and monitored and had access to food and water ad libitum for at least 1 week.

### Recording

The recording was started once mice completely recovered from all surgeries and a normal circadian rhythm was present. A recovery period of more than 7 days was set after surgery. After the recovery period, the dummy fiber was removed, an optical silica fiber (LUCIR, Tsukuba, Japan, Fig. 1E) was inserted, and mice were moved to their measurement cages (Fig. 1F). Locomotor activity was recorded with a passive pyroelectric infrared motion sensor (AMN 1111, Panasonic Co., Osaka, Japan) that was attached to the ceiling of the experimental cage (Fig. 1F). Mice were housed individually in the recording cage during the recording and recovery period which was 1–2 weeks. Orexin neuronal activity and heart rate were recorded for 2–3 successive days using LabChart software version 8 (ADInstruments, New South Wales, Australia) in unrestrained, freely moving, and unanesthetized conditions.

### Fiber photometry device

A fiber photometry system (COME2-FTR/OPT, LUCIR, Tsukuba, Japan) was used to record the activity of orexin neurons in freely moving mice (Fig. 1C). The system utilizes a single silica fiber that can deliver two excitation lights and detect fluorescence from G-CaMP6 and mCherry simultaneously. Blue excitation light for G-CaMP6 (470 nm, 0.5 mW at the tip of the silica fiber) and yellow excitation light for mCherry (590 nm) were produced by a high-power LED system, blue; Thorlabs OPT/LED Blue_TT_FC, yellow; Thorlabs OPT/LED yellow_TT_FC (Thorlabs Japan, Tokyo, Japan). Blue and yellow excitation light was reflected by a dichroic mirror, passed through an excitation bandpass filter, and was delivered via a 400 μm silica fiber into the brain. G-CaMP6 and mCherry fluorescence were collected by the same silica fiber and guided to an individual photomultiplier for either G-CaMP6 or mCherry (PMTH-S1M1-CR131, Zolix instruments, Beijing, China) (Fig. 1F). The signal was digitized using a data acquisition system (PowerLab16/35, ADInstruments, New South Wales, Australia), and recorded by LabChart software version 8. Signals were collected at a sampling frequency of 100 Hz.

### Stress stimulation

Three types of stressors were applied on the following day after the basal measurement was completed (Fig. 2A). The first was a socioemotional stressor via the resident-intruder stress test. This stressor was applied by placing an age-matched wild-type mouse (intruder mouse) contained in a small polypropylene cage into the experimental cage for 2 min. The polypropylene cage is constructed so that the intruder and resident (experimental) mice are unable to physically contact, but visual, auditory, and olfactory communication is available. The second stressor was aversive sound. For this test, an approximately 100 dB/25 kHz ultrasonic sound was applied to the mouse via an ultrasound-emitting device (PET-AGREE, K-II enterprise, New York, USA). The device was turned on above the cage for 2 s. The third stressor was an aversive smell. For the aversive smell, 2,4,5-trimethylthiazoline, (TMT, Contech Enterprise, Victoria, Canada) a constituent of fox urine and feces, a common predator odor for mice [31, 32], was placed near the nose of the mouse for 2 s via cotton swab.

The rationale for choosing these particular types of aversive stimuli was carefully considered. First, intruder stress has been used in orexin knockout mice showing a possible role of orexin neurons in stress-induced autonomic responses [3]. Second, if the sensory component of stress directly activates orexin neurons, there might be a different response depending on sensory modality (optic, auditory, and olfactory) because the information transmitted to the brain from the respective receptors (eyes, ears, and nose) occurs via different sensory afferent pathways.

As the control of stress stimuli, the physiological response was measured using a cotton swab without odor and an empty box without intruders (Fig. 2E).

### Fear conditioning

We made our experimental paradigm with reference to the commonly used fear conditioning tests in mice [33, 34]. The animal was placed in an experimental chamber and acclimatized for 2 h and then a neutral tone sound (60 dB, 1 kHz) was given for 2 s during fiber photometry and the heart rate recording. To associate a neutral tone with an unpleasant experience (Fig. 4A), a 0.4 mA electric shock (0.5 s) was given to the hind paw of the animal 1 to 5 s after a 2 s neutral tone. This was repeated 15 times using a shock generator (CBX-CT) and a cycle timer (CSG-001, Muromachi Kikai, Tokyo, Japan). The intertrial interval was pseudorandomly set at 60 ± 15 s by the cycle timer. After a resting period of 1 h, the same neutral tone was given during fiber photometry and heart rate recording. In the resting period, mice were in a conditioning cage that is in the same place as during conditioning and test-recording. During the post-recording period, freezing behavior was also measured to confirm that classical fear conditioning had been successfully established. Freezing time was visually calculated by observing videotaped animal behavior by an experimenter who was blinded to the experimental group. No electrical stimulus was given to the control animals. All the procedures were performed in one chamber. Both behavioral tests (sound stimulation and fear conditioning) including baseline recording were performed between 12:00 and 18:00.

### Immunohistochemistry

Mice were deeply anesthetized with urethane (2.0 g/kg, i.p.), and transcardially perfused with 20 ml of saline containing 20 unit/ml heparin followed by 20 ml of chilled 4% paraformaldehyde (Wako Pure Chemical Industries, Ltd., Osaka, Japan) in 0.01 M PBS (pH 7.4). The brain was removed, post-fixed in 4% paraformaldehyde solution at 4 °C overnight, and subsequently immersed in PBS at 4 °C for at least 2 days. A series of 40 μm sections were obtained with a vibratome (SuperMicroSlicer Zero1; DOSAKA EM, Kyoto, Japan). For staining, coronal brain sections were immersed in blocking buffer (1% normal horse serum and 0.3% Triton-X in PBS), then incubated with an anti-orexin A goat antibody (SC-8070, Santa Cruz Biotechnology, Inc., Dallas, TX, USA) at 4 °C overnight. The sections were washed with PBS and incubated in a CF647-conjugated anti-goat IgG antibody (20048, Biotium) for 2 h at room temperature. These brain sections were mounted on a slide and imaged on a fluorescence microscope (BZ-9000, Keyence, Osaka, Japan). The primary and secondary antibodies were diluted in blocking buffer or PBS and consisted of anti-orexin A goat antibody used at 1:200 and CF647-conjugated anti-goat IgG antibody at 1:500. We counted G-CaMP6 positive cells (green), mCherry positive cells (red), and anti-orexin A positive cells (far red) in the hypothalamic area where orexin neurons are located (− 1.0 to − 2.0 mm from bregma) using the NIH ImageJ software. We calculated the relative percentage of G-CaMP6 or mCherry positive neurons among orexin neurons by using the average number of anti-orexin A positive neurons as the reference. For counting, we used one out of every four coronal brain slices in an animal.

### Statistical analysis

Statistical analyses were performed using PRISM (GraphPad Software, La Jolla, CA, USA). Simple comparisons of the means between the two groups were performed by Student’s t-test or Mann–Whitney’s nonparametric test. Multiple comparisons of the means and SEM were performed by one-way ANOVA analyses followed by Tukey’s multiple comparison tests, or two-way ANOVA analysis followed by Bonferroni’s multiple comparison tests. A P value of less than 0.05 was considered significant.

## Data Availability

The summary statistics are available within the article. The data that support the findings of this study are available from the corresponding author upon reasonable request.
